# Associations of Dietary Intake of Vitamin B6 and Plasma Pyridoxal 5′‐Phosphate Level With Depression in US Adults: Findings From NHANES 2005–2010

**DOI:** 10.1002/brb3.70128

**Published:** 2024-11-07

**Authors:** Jinhong Lu, Huina Mao, Yulei Tan, Guizhi Luo

**Affiliations:** ^1^ Department of General Surgery Zhujiang Hospital, Southern Medical University Guangzhou China; ^2^ Nursing Department Zhujiang Hospital, Southern Medical University Guangzhou China

**Keywords:** depression, dietary intake of vitamin B6, NHANES, plasma pyridoxal 5′‐phosphate level, US adults

## Abstract

**Background:**

Evidence regarding the associations of pyridoxal 5′‐phosphate level in plasma and dietary intake of vitamin B6 with depression risk is scarce. Accordingly, we investigated the aforementioned associations in US adults.

**Methods:**

This is a cross‐sectional study that included data from two independent samples of 12,716 and 11,967 individuals (aged ≥ 20 years) participating in the National Health and Nutrition Examination Survey (NHANES) from 2005 to 2010. The associations of the pyridoxal 5′‐phosphate level in plasma and dietary intake of vitamin B6 with depression risk were examined through multivariable logistic regression. In addition, we determined dose–response associations by fitting restricted cubic splines to the data.

**Results:**

In the multivariable model, the highest quarter of dietary intake of vitamin B6 was associated with a significantly lower risk of depression compared to the lowest quarter (OR = 0.63, 95% CI: 0.50, 0.79, *p* < 0.001). Similarly, the highest quartile of plasma PLP levels was linked to a reduced risk of depression compared to the lowest quartile (OR = 0.76, 95% CI: 0.62, 0.93, *p* < 0.01). With increasing quartiles of dietary intake of vitamin B6 and plasma PLP levels, the risk of depression also decreased accordingly (all *p* for trend < 0.01). Furthermore, the correlation analysis revealed that for every 1‐SD increase in the level of plasma lutein + zeaxanthin and dietary intake of vitamin B6, the risk of depression showed a decreasing trend (all *p* < 0.01). The interaction test results indicated that the dietary consumption of vitamin B6 did not significantly interact with any of the stratification factors (all *p* for interaction > 0.05). Moreover, no significant interaction was found between the amount of plasma PLP and any hierarchical factors (all *p* for interaction > 0.05), except for gender‐based subgroup analysis (*p* for interaction > 0.05). The dose‐response relationship results showed a linear decrease trend in the relationship between dietary vitamin B6 intake and plasma pyridoxal 5′‐phosphate with the risk of depression.

**Conclusions:**

Plasma PLP levels and dietary vitamin B6 intake in the highest quartiles are associated with a lower risk of depression. These findings support the promotion of a balanced diet rich in vitamin B6. However, future randomized controlled trials are necessary to confirm the effects of vitamin B6 supplementation on depression risk. We should aim for a healthy and balanced diet in terms of nutritional supplementation.

## Graphical abstract

The intake of vitamin B6 in the diet seems to be related to the occurrence of depression. This relationship is not simply linear, but reveals different effects of intake at certain levels through restrictive splines (RCS). Specifically, a negative linear coefficient (−0.9954) indicates that as vitamin B6 intake increases, the risk of depression decreases within a certain range, but this decrease is not a linear decline. The nonlinear coefficient (0.9200) indicates that after a certain intake, the decrease in depression risk may tend to stabilize or reverse, that is, excessive intake of vitamin B6 may no longer continue to reduce depression risk, and may even increase. This result indicates that the relationship between plasma pyridoxal phosphate (PLP) levels and depression is nonlinear. Although an increase in PLP levels is slightly negatively correlated with the risk of depression, the effect is very small (linear coefficient of −0.02734), and at higher levels of PLP, the risk may slightly increase (nonlinear coefficient of 0.03954).

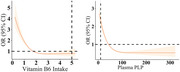

## 1 Introduction

Depression constitutes a widespread emotional disorder that is known to impair a person's psychosocial function; it also lowers their overall quality of life (Thapar et al. 2022). Major depression ranked in 2008 third among the prominent contributors to the global disease burden and is expected to peak in terms of its contribution by 2030 (Malhi and Mann 2018; Vos et al. 2020). In the United States, one‐sixth of people experience depression in their daily life. As shown by epidemiological studies, depression prevalence is higher among young people than other groups, and one‐sixth of men and one‐quarter of women experience depression at some stage (Singhal and Baune 2017). Research demonstrated that in 2019, 7.8% of American adults had at least one severe depressive episode (Substance Abuse and Mental Health Services Administration 2020). In clinical practice, associations have been found between many chronic diseases and depression, and the disease progresses more on average in those with depression than those without (Hu et al. 2023; Shen and Zou 2024). Because of its high prevalence, severe disease burden, and other decisive features, depression has become a severe health issue and a key public health issue globally.

Depression is not engendered by a single mechanism but is engendered by the intricate interaction of biological, social, and psychological factors. From the biological perspective, the synthesis of certain neurotransmitters depends on decarboxylase, for which vitamin B6 is a cofactor (De Souza et al. 2000). Research has shown that depression can be regulated through diet (Opie et al. 2017). Vitamin B6, a water‐soluble vitamin, plays a crucial role in various biological functions, including neurotransmitter synthesis, homocysteine metabolism, and immune function. Previous studies have suggested that vitamin B6 may help modulate inflammation and homocysteine levels, both of which are implicated in the pathophysiology of depression (Dai et al. 2021; Muhamad et al. 2023; Ueland et al. 2017; Zhu et al. 2023). Pyridoxal 5′‐phosphate (PLP) acts as a coenzyme in the synthesis of serotonin, dopamine, and gamma‐aminobutyric acid, neurotransmitters that are closely linked to mood regulation (Ntona et al. 2023; Paulose et al. 1988). Deficiency in vitamin B6 has been associated with an increased risk of mood disorders, including depression (Noah et al. 2021; Tsujita et al. 2019). The vitamin B6 derivative PLP can be obtained from various foods, including meat, milk products, beans, nuts, potatoes, and several fruits and vegetables (Alsaeedi, Welham, and Rose 2023). One study discovered a negative correlation between PLP levels in plasma with systemic biomarkers of inflammation in American adults (Sakakeeny et al. 2012). Few studies have comprehensively explored whether PLP's plasma concentration and dietary intake of vitamin B6 are associated with depression. One cross‐sectional study demonstrated women taking in more dietary vitamin B6 exhibited a lower depression risk (Kafeshani et al. 2020), but the study was conducted in only one country, Iran. Research on the specific relationship between dietary vitamin B6 intake, plasma PLP levels, and depression risk remains limited, particularly in large population‐based studies. No research on the US population has yet investigated the association of depression risk with PLP's plasma concentration and dietary intake of vitamin B6. In addition, scholars have yet to probe whether depression risk and PLP's plasma concentration are linearly related.

With the purpose of filling the aforementioned gaps, we executed our research to probe whether PLP's plasma concentration and dietary intake of vitamin B6 are linked to depression risk in US adults by analyzing data obtained from the National Health and Nutrition Examination Survey (NHANES) 2005–2010 implemented by the Centers for Disease Control and Prevention's National Center for Health Statistics.

## 2 Methods

### 2.1 Population

**FIGURE 1:**
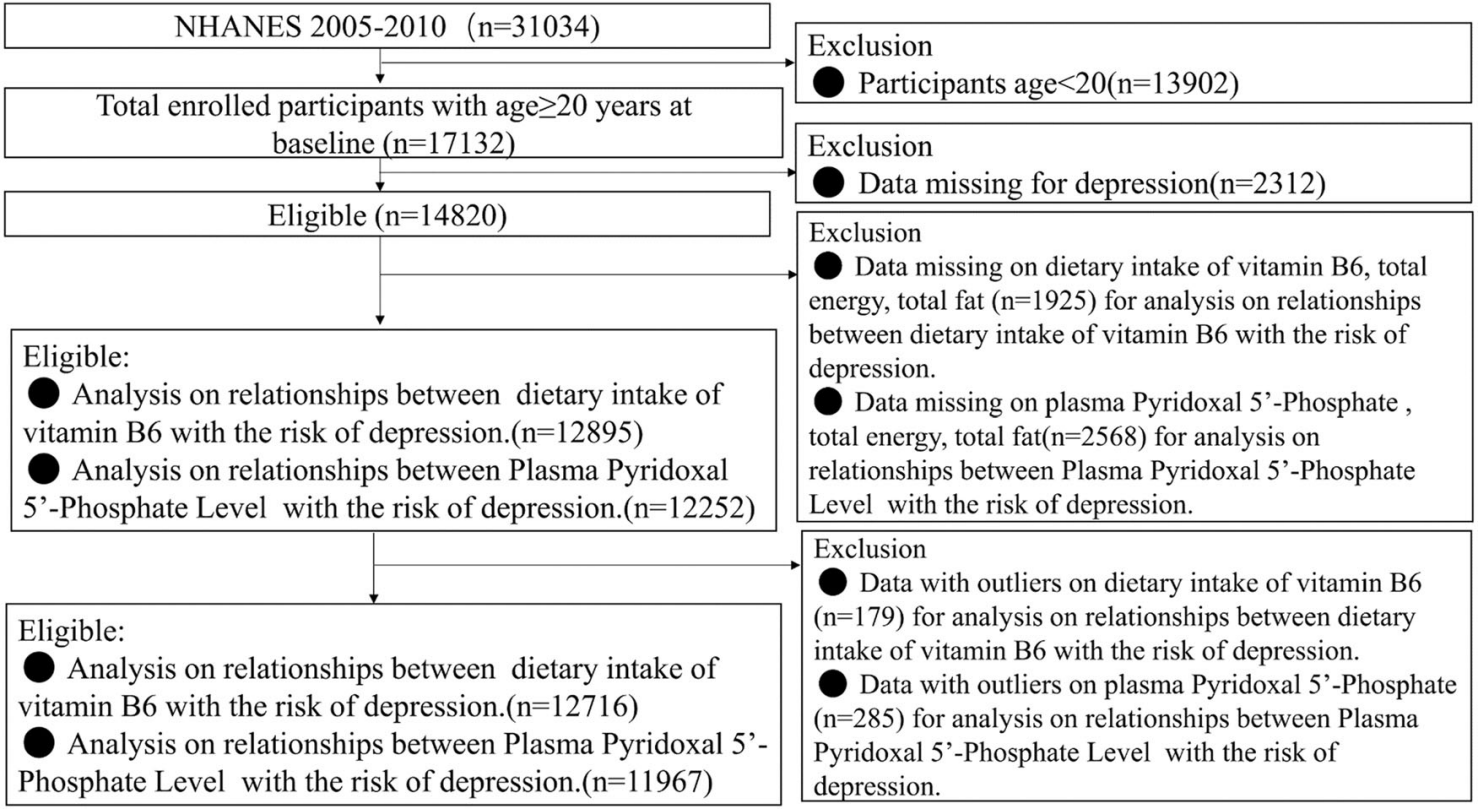
Flow chart of participant selection for the study population, NHANES 2005–2010.

Figure 1 shows the sample selection process in the form of a flowchart. The study used all participants from the NHANES 2005–2010 cycles. The NHANES 2005–2010 involved 31,034 individuals, and we employed data for 17,132 individuals aged 20 years or older. Of these individuals, we excluded 2312 due to incomplete depression data. The association of depression risk with the amount of PLP in plasma and the association of depression risk with the dietary intake of vitamin B6 were analyzed separately. We excluded 2568 and 1925 individuals with missing data, respectively. Subsequently, we removed 285 and 179 participants with outliers, respectively. Ultimately, 11,967 and 12,716 individuals were included in the analyses, respectively. Following the Declaration of Helsinki, all individuals participating in the NHANES gave their written informed consent. In addition, the research ethics review board overseeing the operations of the National Center for Health Statistics ratified the NHANES protocols.

### 2.2 Dietary Intake of Vitamin B6 and PLP Level in Plasma Measurement

The participating individuals' dietary intake of vitamin B6 was measured through the 24‐h recall method involving a dietary recall interview in the NHANES. Two days of data on dietary intake were collected by trained staff. First, each participant took part in an in‐person interview that was executed at a mobile examination center to collect the first day's data; subsequently, 3–10 days later, they took part in a telephone interview that was executed to collect the second day's data. The retention factor recipe calculation method is employed to evaluate the nutrient value of food mixtures, whereas label information is used for evaluations of commercial mixtures. Furthermore, a set of retention factors is used in the FNDDS to account for the nutrients that are lost from food during cooking processes. In this research, two dietary recall interviews were conducted to evaluate dietary intake of vitamin B6, total energy, and total fat. Our analysis of dietary intake was based on the average of two 24‐h dietary recall interviews conducted by the NHANES.

The detailed laboratory procedure used for measuring a participant's plasma PLP level (nmol/L) is provided in the NHANES documentation. In brief, an enzymatic assay kit characterized by homogeneity and nonradioactivity (A/C Diagnostics, San Diego, CA, USA) was employed. The mean inter‐assay and intra‐assay coefficients of variation were 12.0%–13.1% and 7.8%–8.3%, respectively. Assay values that were lower than the limit of detection were higher than 7.1 nmol/L (which is 10.09 nmol/L, the limit of detection, divided by the square root of 2).

### 2.3 Outcome Definition

In the NHANES, depression was diagnosed in accordance with the self‐reported Patient Health Questionnaire (PHQ‐9), which is a tool for assessing depressive symptoms (Kroenke, Spitzer, and Williams 2001). When the participants completed PHQ‐9, they were asked about their depressive symptoms over the preceding fortnight. The questionnaire contains items on nine symptoms, and the participants responded to the items on a 4‐point scale with the following points: 0 (“*not at all*”), 1 (“*several days*”), 2 (“*more than half the days*”), and 3 (“*nearly every day*”). The total score, obtained by summing the scores for all items, ranges from 0 to 27. We considered a participant to have depression when their total PHQ‐9 score was ≥ 10, as outlined by other research (Ba et al. 2021; Wang et al. 2016).

### 2.4 Statistical Analysis

We describe normally distributed continuous and categorical variables in terms of means plus standard deviations (SDs) and numbers and percentages, respectively. Analysis of variance was employed to determine intermean differences and categorical variable comparisons was executed by employing a chi‐square test. In addition, through multiple logistic regression analysis, we estimated the associations of depression risk with plasma PLP levels and dietary intake of vitamin B6 by deriving odds ratios (OR) and their corresponding 95% confidence intervals (CIs). Covariates were selected based on their impact on the OR values. Following a standard approach, we included covariates that resulted in a change of more than 10% in OR values, indicating a significant influence on the relationship between the independent and dependent variables. This approach is widely accepted in statistical analysis. Furthermore, we considered previous literature to include covariates that have been shown to influence depression risk, such as age, gender, leisure‐time physical activity, maritial status and so on. We also derived multivariable models for which we adjusted for the following variables: ratio of family income to the poverty level (< 2.34, ≥ 2.34, or missing), dietary intake of total energy (unit, kcal; continuous), dietary intake of total fat (unit, g; continuous), in addition to ethnicity (Mexican American, non‐Hispanic White, Black, other Hispanic, or other ethnicity), gender (male or female), educational level (high school or lower, high school, college graduate or higher, or missing), marital status ((married, widowed, divorced, separated, living with partner, never married or missing), body mass index (BMI; unit, kg/m^2^; < 25, ≥ 25, or missing), age (continuous), smoking status (yes, no, or missing), alcohol status (yes, no), leisure‐time physical activity (unit: metabolic equivalents/week; < 500, 500–< 1000, ≥ 1000, or missing), hypertension(yes, no, or missing), diabetes(yes, no, or missing), coronary heart disease (yes, no, or missing), and sleep disorder (yes, no, or missing). In addition, through multiple logistic regression analysis, we estimated the associations of depression risk with plasma PLP levels and dietary intake of vitamin B6. Both variables were divided into quartiles to facilitate comparisons, as commonly done in NHANES‐based studies. Quartiles are a commonly used approach in NHANES data analysis to facilitate comparisons across different levels of exposure. We next fit restricted cubic splines to the data derived for the dose–response associations of depression risk with the PLP level in plasma and dietary intake of vitamin B6. R software 3.6.2 served as the platform for all of our executed statistical analyses. Moreover, we considered a two‐sided *p* < 0.05 as indicating statistical significance.

## 3 Results

### 3.1 Participant Characteristics

Tables 1, 2, 3, 4 show the general characteristics of the study population, presented in quartiles of dietary vitamin B6 intake and plasma PLP level. The included NHANES 2005–2010 participants were divided into four groups on the basis of the quartiles (Q1–Q4) of their plasma levels of PLP and four groups on the basis of the quartiles (Q1–Q4) of their dietary intake of vitamin B6 (Table 1). The total number of individuals in the plasma PLP groups was 11,967 (5762 men and 6205 women), and that of those in the four dietary vitamin B6 intake groups was 12,716 (6064 men and 6652 women); the mean ages in these two group sets were 49.87 ± 17.89 and 50.06 ± 17.95 years, respectively. Dietary vitamin B6 intake was higher in men, younger individuals, those with higher family income, those who achieved a college graduate or above education level, those with a lower BMI, those with more exercise leisure time, those consuming more fat, and those consuming more energy. The PLP level in plasma was higher in those with higher family income, achieved a college graduate or above education level, lower BMI, and more exercise leisure time.

**TABLE 1:** Univariate analyses of socioeconomic, lifestyle factors, physical condition, and dietary information with depression, presented by dietary vitamin B6 intake.

	Nondepression	Depression	*p* value
*N*	11,621	1095	
Age, years	50.28 ± 18.17	47.80 ± 15.39	< 0.001
Gender			< 0.001
Male	5669 (48.78%)	395 (36.07%)	
Female	5952 (51.22%)	700 (63.93%)	
Race			< 0.001
Non‐Hispanic White	5955 (51.24%)	491 (44.84%)	
Black	2244 (19.31%)	236 (21.55%)	
Other Hispanic	923 (7.94%)	127 (11.60%)	
Mexican American	2057 (17.70%)	198 (18.08%)	
Other race/ethnicity	442 (3.80%)	43 (3.93%)	
Education level			< 0.001
Less than high school	1269 (10.93%)	178 (16.26%)	
High school	4524 (38.97%)	523 (47.76%)	
College graduate or above	5816 (50.10%)	394 (35.98%)	
Marital status			< 0.001
Married	6526 (56.19%)	441 (40.27%)	
Widowed	991 (8.53%)	91 (8.31%)	
Divorced	1172 (10.09%)	178 (16.26%)	
Separated	312 (2.69%)	79 (7.21%)	
Never married	1771 (15.25%)	193 (17.63%)	
Living with partner	842 (7.25%)	113 (10.32%)	
The ratio of family income to poverty	2.69 ± 1.61	1.76 ± 1.41	< 0.001
BMI, kg/m^2^	28.96 ± 6.51	30.70 ± 8.11	< 0.001
Leisure exercise time, MET/week	927.17 ± 1784.03	471.22 ± 1452.77	< 0.001
Drinking			< 0.001
No	8660 (74.52%)	901 (82.28%)	
Yes	2961 (25.48%)	194 (17.72%)	
Smoking status			< 0.001
Never	6276 (54.04%)	438 (40.00%)	
Past	3107 (26.75%)	228 (20.82%)	
Current	2231 (19.21%)	429 (39.18%)	
Hypertension			< 0.001
No	7605 (65.55%)	593 (54.20%)	
Yes	3997 (34.45%)	501 (45.80%)	
Diabetes			< 0.001
No	10,090 (88.48%)	870 (81.69%)	
Yes	1314 (11.52%)	195 (18.31%)	
Coronary heart disease			< 0.05
No	11,098 (95.87%)	1028 (94.31%)	
Yes	478 (4.13%)	62 (5.69%)	
Sleep disorders			< 0.001
Yes	2481 (21.36%)	593 (54.16%)	
No	9136 (78.64%)	502 (45.84%)	
Energy (kcal)	2026.61 ± 808.39	1901.26 ± 801.85	< 0.001
Total fat (g)	75.83 ± 37.18	70.40 ± 36.16	< 0.001

*Note*: MET (metabolic equivalent of task) represents the amount of energy expended during physical activity. One MET is the amount of energy expended while sitting quietly. MET/week refers to the metabolic equivalents expended through physical activity in a week.

**TABLE 2:** Univariate analyses of socioeconomic, lifestyle factors, physical condition, and dietary information with depression, presented by plasma pyridoxal 5′‐phosphate.

	Nondepression	depression	*p* value
*N*	10,933	1034	
Age, years	50.10 ± 18.11	47.53 ± 15.30	< 0.001
Gender			< 0.001
Male	5382 (49.23%)	380 (36.75%)	
Female	5551 (50.77%)	654 (63.25%)	
Race			< 0.001
Non‐Hispanic White	5649 (51.67%)	470 (45.45%)	
Black	2061 (18.85%)	219 (21.18%)	
Other Hispanic	871 (7.97%)	122 (11.80%)	
Mexican American	1946 (17.80%)	181 (17.50%)	
Other race/ethnicity	406 (3.71%)	42 (4.06%)	
Education level			< 0.001
Less than high school	1194 (10.93%)	168 (16.25%)	
High school	4266 (39.06%)	491 (47.49%)	
College graduate or above	5463 (50.01%)	375 (36.27%)	
Marital status			< 0.001
Married	6123 (56.03%)	414 (40.04%)	
Widowed	927 (8.48%)	82 (7.93%)	
Divorced	1101 (10.08%)	168 (16.25%)	
Separated	300 (2.75%)	75 (7.25%)	
Never married	1670 (15.28%)	185 (17.89%)	
Living with partner	807 (7.38%)	110 (10.64%)	
The ratio of family income to poverty	2.69 ± 1.61	1.78 ± 1.42	< 0.001
BMI, kg/m^2^	28.99 ± 6.53	30.79 ± 8.09	< 0.001
Leisure exercise time, MET/week	925.87 ± 1776.46	513.45 ± 1539.01	< 0.001
Drinking			< 0.001
No	8138 (74.44%)	843 (81.53%)	
Yes	2795 (25.56%)	191 (18.47%)	
Smoking status			< 0.001
Never	5877 (53.79%)	412 (39.85%)	
Past	2894 (26.49%)	218 (21.08%)	
Current	2155 (19.72%)	404 (39.07%)	
Hypertension			< 0.001
No	7194 (65.91%)	564 (54.60%)	
Yes	3721 (34.09%)	469 (45.40%)	
Diabetes			< 0.001
No	9506 (88.62%)	823 (81.97%)	
Yes	1221 (11.38%)	181 (18.03%)	
Coronary heart disease			0.069
No	10,440 (95.85%)	974 (94.66%)	
Yes	452 (4.15%)	55 (5.34%)	
Sleep disorders			< 0.001
Yes	2323 (21.26%)	555 (53.68%)	
No	8606 (78.74%)	479 (46.32%)	
Energy (kcal)	2043.95 ± 821.87	1924.71 ± 834.94	< 0.001
Total fat (g)	76.39 ± 37.59	71.48 ± 38.26	< 0.001

**TABLE 3:** General characteristics of study population, presented in quartiles of dietary vitamin B6 intake.

Vitamin B6 intake (mg)	Q1 (0.1305–1.2655)	Q2 (1.2660–1.7515)	Q3 (1.7520–2.4005)	Q4 (2.4010–5.3440)	*p* value
*N*	3177	3180	3177	3182	
Age, years	51.77 ± 18.34	50.32 ± 17.87	50.13 ± 17.87	48.04 ± 17.55	< 0.001
Gender					< 0.001
Male	976 (30.72%)	1293 (40.66%)	1649 (51.90%)	2146 (67.44%)	
Female	2201 (69.28%)	1887 (59.34%)	1528 (48.10%)	1036 (32.56%)	
Race					< 0.001
Non‐Hispanic White	1463 (46.05%)	1536 (48.30%)	1643 (51.72%)	1804 (56.69%)	
Black	735 (23.14%)	638 (20.06%)	603 (18.98%)	504 (15.84%)	
Other Hispanic	299 (9.41%)	286 (8.99%)	261 (8.22%)	204 (6.41%)	
Mexican American	552 (17.37%)	600 (18.87%)	545 (17.15%)	558 (17.54%)	
Other race/ethnicity	128 (4.03%)	120 (3.77%)	125 (3.93%)	112 (3.52%)	
Education level					< 0.001
Less than high school	509 (16.04%)	375 (11.81%)	311 (9.80%)	252 (7.92%)	
High school	1395 (43.96%)	1257 (39.59%)	1214 (38.25%)	1181 (37.12%)	
College graduate or above	1269 (39.99%)	1543 (48.60%)	1649 (51.95%)	1749 (54.97%)	
Marital status					< 0.001
Married	1548 (48.74%)	1744 (54.88%)	1834 (57.75%)	1841 (57.91%)	
Widowed	365 (11.49%)	280 (8.81%)	250 (7.87%)	187 (5.88%)	
Divorced	415 (13.07%)	338 (10.64%)	308 (9.70%)	289 (9.09%)	
Separated	122 (3.84%)	96 (3.02%)	89 (2.80%)	84 (2.64%)	
Never married	506 (15.93%)	486 (15.29%)	456 (14.36%)	516 (16.23%)	
Living with partner	220 (6.93%)	234 (7.36%)	239 (7.53%)	262 (8.24%)	
The ratio of family income to poverty	2.28 ± 1.55	2.60 ± 1.59	2.72 ± 1.62	2.83 ± 1.64	< 0.001
BMI, kg/m^2^	29.47 ± 7.01	29.33 ± 6.75	29.17 ± 6.71	28.47 ± 6.21	< 0.001
Leisure time, MET/week	701.33 ± 1577.20	766.82 ± 1576.73	884.16 ± 1612.94	1193.14 ± 2161.23	< 0.001
Drinking					< 0.001
No	2704 (85.11%)	2497 (78.52%)	2283 (71.86%)	2077 (65.27%)	
Yes	473 (14.89%)	683 (21.48%)	894 (28.14%)	1105 (34.73%)	
Smoking status					< 0.001
Never	1609 (50.69%)	1754 (55.16%)	1712 (53.89%)	1639 (51.57%)	
Past	745 (23.47%)	823 (25.88%)	854 (26.88%)	913 (28.73%)	
Current	820 (25.83%)	603 (18.96%)	611 (19.23%)	626 (19.70%)	
Hypertension					< 0.001
No	1909 (60.20%)	2002 (63.07%)	2086 (65.78%)	2201 (69.21%)	
Yes	1262 (39.80%)	1172 (36.93%)	1085 (34.22%)	979 (30.79%)	
Diabetes					< 0.001
No	2648 (85.23%)	2689 (86.30%)	2771 (88.87%)	2852 (91.18%)	
Yes	459 (14.77%)	427 (13.70%)	347 (11.13%)	276 (8.82%)	
Coronary heart disease					0.662
No	3008 (95.37%)	3042 (95.99%)	3033 (95.80%)	3043 (95.78%)	
Yes	146 (4.63%)	127 (4.01%)	133 (4.20%)	134 (4.22%)	
Sleep disorders					< 0.001
Yes	875 (27.56%)	795 (25.01%)	715 (22.51%)	689 (21.65%)	
No	2300 (72.44%)	2384 (74.99%)	2461 (77.49%)	2493 (78.35%)	
Depression					< 0.001
No	2774 (87.32%)	2894 (91.01%)	2962 (93.23%)	2991 (94.00%)	
Yes	403 (12.68%)	286 (8.99%)	215 (6.77%)	191 (6.00%)	
Energy (kcal)	1419.04 ± 499.61	1820.80 ± 549.79	2149.58 ± 634.33	2672.99 ± 903.69	< 0.001
Total fat (g)	53.58 ± 24.10	68.80 ± 28.21	80.51 ± 32.71	98.52 ± 44.56	< 0.001

**TABLE 4:** General characteristics of study population, presented in quartiles of plasma pyridoxal 5′‐phosphate level.

Plasma PLP (nmol/L)	Q1 (2.00–25.80)	Q2 (25.90–42.80)	Q3 (42.90–73.90)	Q4 (74.00–324.00)	*p* value
*N*	2989	2988	2995	2995	
Age, years	51.68 ± 17.34	48.50 ± 17.65	48.19 ± 17.99	51.13 ± 18.34	< 0.001
Gender					< 0.001
Male	1149 (38.44%)	1415 (47.36%)	1646 (54.96%)	1552 (51.82%)	
Female	1840 (61.56%)	1573 (52.64%)	1349 (45.04%)	1443 (48.18%)	
Race					< 0.001
Non‐Hispanic White	1461 (48.88%)	1409 (47.16%)	1487 (49.65%)	1762 (58.83%)	
Black	765 (25.59%)	579 (19.38%)	485 (16.19%)	451 (15.06%)	
Other Hispanic	213 (7.13%)	288 (9.64%)	275 (9.18%)	217 (7.25%)	
Mexican American	473 (15.82%)	598 (20.01%)	618 (20.63%)	438 (14.62%)	
Other race/ethnicity	77 (2.58%)	114 (3.82%)	130 (4.34%)	127 (4.24%)	
Education level					< 0.001
Less than high school	390 (13.06%)	373 (12.49%)	337 (11.27%)	262 (8.75%)	
High school	1431 (47.92%)	1239 (41.48%)	1122 (37.53%)	965 (32.23%)	
College graduate or above	1165 (39.02%)	1375 (46.03%)	1531 (51.20%)	1767 (59.02%)	
Marital status					< 0.001
Married	1448 (48.44%)	1587 (53.17%)	1726 (57.65%)	1776 (59.32%)	
Widowed	319 (10.67%)	232 (7.77%)	200 (6.68%)	258 (8.62%)	
Divorced	404 (13.52%)	326 (10.92%)	259 (8.65%)	280 (9.35%)	
Separated	131 (4.38%)	99 (3.32%)	73 (2.44%)	72 (2.40%)	
Never married	445 (14.89%)	482 (16.15%)	497 (16.60%)	431 (14.40%)	
Living with partner	242 (8.10%)	259 (8.68%)	239 (7.98%)	177 (5.91%)	
The ratio of family income to poverty	2.19 ± 1.51	2.50 ± 1.59	2.76 ± 1.63	2.99 ± 1.62	< 0.001
BMI, kg/m^2^	31.01 ± 8.01	29.44 ± 6.70	28.55 ± 5.85	27.61 ± 5.49	< 0.001
Leisure exercise time, MET/week	623.25 ± 1594.95	794.80 ± 1626.06	967.28 ± 1726.92	1155.03 ± 2006.72	< 0.001
Drinking					< 0.001
No	2549 (85.28%)	2270 (75.97%)	2074 (69.25%)	2088 (69.72%)	
Yes	440 (14.72%)	718 (24.03%)	921 (30.75%)	907 (30.28%)	
Smoking status					< 0.001
Never	1332 (44.58%)	1533 (51.37%)	1668 (55.69%)	1756 (58.67%)	
Past	697 (23.33%)	725 (24.30%)	839 (28.01%)	851 (28.43%)	
Current	959 (32.10%)	726 (24.33%)	488 (16.29%)	386 (12.90%)	
Sleep disorders					< 0.001
Yes	869 (29.10%)	641 (21.45%)	641 (21.40%)	727 (24.28%)	
No	2117 (70.90%)	2347 (78.55%)	2354 (78.60%)	2267 (75.72%)	
Hypertension					< 0.001
No	1740 (58.29%)	1975 (66.30%)	2048 (68.47%)	1995 (66.66%)	
Yes	1245 (41.71%)	1004 (33.70%)	943 (31.53%)	998 (33.34%)	
Diabetes					< 0.001
No	2450 (83.50%)	2587 (88.29%)	2624 (89.59%)	2668 (90.81%)	
Yes	484 (16.50%)	343 (11.71%)	305 (10.41%)	270 (9.19%)	
Coronary heart disease					0.002
No	2821 (94.79%)	2851 (95.77%)	2884 (96.78%)	2858 (95.65%)	
Yes	155 (5.21%)	126 (4.23%)	96 (3.22%)	130 (4.35%)	
Energy (kcal)	1895.99 ± 792.83	2024.47 ± 841.26	2113.01 ± 837.79	2100.82 ± 803.89	< 0.001
Total fat (g)	72.41 ± 36.57	76.25 ± 38.78	78.29 ± 38.54	76.90 ± 36.48	< 0.001
Depression					< 0.001
No	2599 (86.95%)	2714 (90.83%)	2799 (93.46%)	2821 (94.19%)	
Yes	390 (13.05%)	274 (9.17%)	196 (6.54%)	174 (5.81%)	

### 3.2 Multivariate Logistic Regression Analysis for Associations of Depression Risk With Plasma Level of PLP and Dietary Intake of Vitamin B6

**FIGURE 2:**
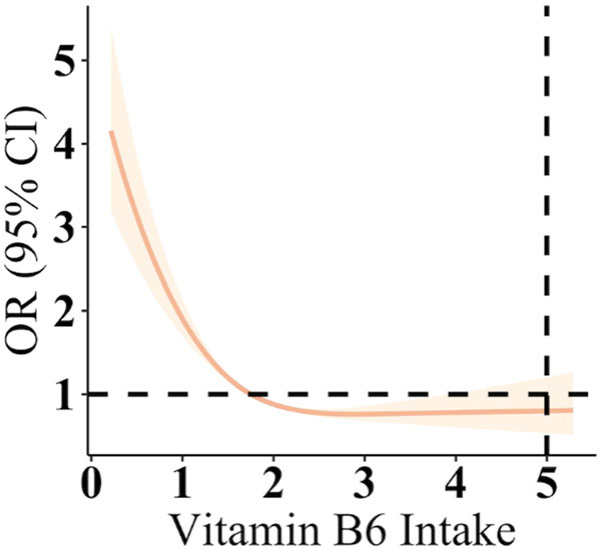
Dose–response association between dietary vitamin B6 intake and depression risk.

**FIGURE 3:**
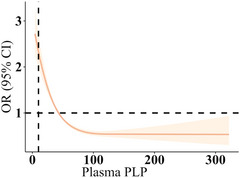
Dose–response association between plasma pyridoxal 5′‐phosphate level and depression risk.

The unadjusted model revealed that depression was negatively associated with both the PLP level in plasma and dietary intake of vitamin B6 (Table 5). Dietary intake of vitamin B6 at the Q4 level (highest intake) was noted to be associated with significantly lower depression risk than that at the Q1 level (OR = 0.44; 95% CI: 0.37, 0.53; *p *< 0.001). In addition, plasma PLP concentration at the Q4 level (highest level) was associated with significantly lower depression risk than that at the Q1 level (OR = 0.41; 95% CI: 0.34, 0.50; *p *< 0.001). In Adjusted Model I—in which we adjusted for marital status, gender, ethnicity, age, and educational level—we noted depression risk to exhibit negative associations with the PLP level in plasma and intake of dietary vitamin B6. Dietary intake of vitamin B6 at the Q4 level was associated with significantly lower depression risk than that at the Q1 level (OR = 0.56; 95% CI: 0.46, 0.68; *p* < 0.001), as was the Q4 plasma PLP concentration compared with the Q1 level (OR = 0.51; 95% CI: 0.42, 0.52; *p* < 0.001). The negative associations remained in Adjusted Model II, in which we controlled for additional factors to those in Adjusted Model I, namely BMI, smoking status, exercise leisure time, sleep disorders, alcohol status, total energy intake, total fat intake, coronary heart disease, hypertension, and diabetes, as well as the ratio of family income to the poverty level. Dietary intake of vitamin B6 at the Q4 level was associated with significantly lower depression risk than that at the Q1 level (OR = 0.63; 95% CI: 0.50, 0.79; *p* < 0.001). The PLP level in plasma at the Q4 level was associated with significantly lower depression risk than that at the Q1 level (OR = 0.76; 95% CI: 0.62, 0.93; *p* < 0.01). With increasing quartiles of dietary intake of vitamin B6 and plasma PLP levels, the risk of depression also decreased accordingly (all *p* for trend < 0.01). Furthermore, the correlation analysis revealed that for every 1‐SD increase in the level of plasma lutein + zeaxanthin and dietary intake of vitamin B6, the risk of depression showed a decreasing trend (all *p* < 0.01). As shown in Figures 2 and 3, the association between dietary vitamin B6 intake and plasma PLP with the risk of depression showed a linear downward trend.

**TABLE 5:** Associations between dietary intake of vitamin B6 and plasma pyridoxal 5′‐phosphate level with depression.

Exposure	Nonadjusted		Adjust I		Adjust II	
	Odds ratio (95% CI)	*p* value	Odds ratio (95% CI)	*p* value	Odds ratio (95% CI)	*p* value
Vitamin B6 Intake(mg)						
Per 1 SD increase in Vitamin B6	0.71 (0.66, 0.77)	<0.001	0.79 (0.73, 0.85)	< 0.001	0.82 (0.75, 0.90)	< 0.001
Q1	Reference (1.00)		Reference (1.00)		Reference (1.00)	
Q2	0.68 (0.58, 0.80)	< 0.001	0.76 (0.65, 0.90)	< 0.01	0.81 (0.67, 0.96)	< 0.05
Q3	0.50 (0.42, 0.59)	< 0.001	0.60 (0.50, 0.71)	< 0.001	0.66 (0.54, 0.81)	< 0.001
Q4	0.44 (0.37, 0.53)	< 0.001	0.56 (0.46, 0.68)	< 0.001	0.63 (0.50, 0.79)	< 0.001
*p* for trend	< 0.001		< 0.001		< 0.001	
Plasma PLP (nmol/L)						
Per 1 SD increase in Plasma PLP	0.69 (0.63, 0.76)	< 0.001	0.76 (0.70, 0.83)	< 0.001	0.89 (0.82, 0.97)	< 0.01
Q1	Reference (1.00)		Reference (1.00)		Reference (1.00)	
Q2	0.67 (0.57, 0.79)	< 0.001	0.72 (0.61, 0.85)	< 0.001	0.91 (0.76, 1.09)	0.301
Q3	0.47 (0.39, 0.56)	< 0.001	0.54 (0.45, 0.65)	< 0.001	0.76 (0.62, 0.92)	< 0.01
Q4	0.41 (0.34, 0.50)	< 0.001	0.51 (0.42, 0.62)	< 0.001	0.76 (0.62, 0.93)	< 0.01
*p* for trend	< 0.001		< 0.001		< 0.01	

*Note*: Nonadjusted model adjust for: None. Adjust I model adjust for: age, gender, race, education level, marital status. Adjust II model adjust for: age, gender, race, education level, marital status, BMI, smoking status, free exercise time, the ratio of family income to poverty, sleep disorders, total energy, total fat, hypertension, diabetes, coronary heart disease, drinking status.

The intake of vitamin B6 in the diet seems to be related to the occurrence of depression. This relationship is not simply linear, but reveals different effects of intake at certain levels through restrictive splines (RCS). Specifically, a negative linear coefficient (−0.9954) indicates that as vitamin B6 intake increases, the risk of depression decreases within a certain range, but this decrease is not a linear decline. The nonlinear coefficient (0.9200) indicates that after a certain intake, the decrease in depression risk may tend to stabilize or reverse, that is, excessive intake of vitamin B6 may no longer continue to reduce depression risk and may even increase.

This result indicates that the relationship between plasma PLP (pyridoxal phosphate) levels and depression is nonlinear. Although an increase in PLP levels is slightly negatively correlated with the risk of depression, the effect is very small (linear coefficient of −0.02734), and at higher levels of PLP, the risk may slightly increase (nonlinear coefficient of 0.03954).

### 3.3 Subgroup Analyses

Tables 6 and 7 detail the associations of depression risk with the dietary intake of vitamin B6 and plasma level of PLP, respectively, in different subgroups defined on the basis of age, gender, marital status, educational level, ethnicity, and BMI, as well as the ratio of family income to the poverty level.

**TABLE 6:** Association between dietary vitamin B6 intake and depression in different subgroups.

	Vitamin B6 intake (mg)					
	Q1 (0.1305–1.2655)	**Q2 (1.2660–1.7515)**	**Q3 (1.7520–2.4005)**	**Q4 (2.4010–5.3440)**	*p* for trend	*p* for interaction
Age						0.323
< 55	Reference (1.00)	0.83 (0.66, 1.04) 0.102	0.66 (0.52, 0.86) < 0.01	0.58 (0.44, 0.78) < 0.001	< 0.001	
≥ 55	Reference (1.00)	0.74 (0.55, 1.00) 0.052	0.62 (0.43, 0.87) < 0.01	0.72 (0.48, 1.10) 0.127	< 0.05	
Gender						0.597
Male	Reference (1.00)	0.87 (0.63, 1.21) 0.411	0.61 (0.43, 0.86) < 0.01	0.65 (0.45, 0.95) < 0.05	< 0.01	
Female	Reference (1.00)	0.76 (0.61, 0.94) < 0.05	0.67 (0.52, 0.86) < 0.01	0.59 (0.43, 0.81) < 0.01	< 0.001	
Race						0.231
Non‐Hispanic White	Reference (1.00)	0.82 (0.63, 1.07) 0.139	0.55 (0.40, 0.75) < 0.001	0.62 (0.45, 0.87) < 0.01	< 0.001	
Others	Reference (1.00)	0.79 (0.62, 1.00) 0.054	0.75 (0.57, 0.98) < 0.05	0.63 (0.45, 0.89) < 0.01	< 0.01	
Education level						0.670
Less than high school	Reference (1.00)	0.81 (0.53, 1.26) 0.356	0.56 (0.32, 0.97) < 0.05	0.67 (0.35, 1.28) 0.225	0.084	
High school	Reference (1.00)	0.78 (0.60, 1.02) 0.070	0.62 (0.46, 0.83) < 0.01	0.67 (0.48, 0.93) < 0.05	< 0.01	
College graduate or above	Reference (1.00)	0.83 (0.62, 1.12) 0.227	0.74 (0.53, 1.03) 0.072	0.58 (0.39, 0.86) < 0.01	< 0.01	
Marital status						0.613
Married/living with partner	Reference (1.00)	0.80 (0.63, 1.03) 0.086	0.60 (0.45, 0.80) < 0.001	0.59 (0.43, 0.82) < 0.01	< 0.001	
Widowed/divorced/separated	Reference (1.00)	0.76 (0.55, 1.05) 0.095	0.72 (0.50, 1.04) 0.077	0.73 (0.47, 1.12) 0.152	0.089	
Never married	Reference (1.00)	0.87 (0.56, 1.33) 0.510	0.75 (0.46, 1.21) 0.238	0.59 (0.33, 1.04) 0.067	0.063	
The ratio of family income to poverty						0.308
< 2.34	Reference (1.00)	0.75 (0.60, 0.93) < 0.01	0.59 (0.46, 0.75) < 0.001	0.61 (0.45, 0.81) < 0.001	< 0.001	
≥ 2.34	Reference (1.00)	1.04 (0.72, 1.51) 0.820	0.91 (0.61, 1.36) 0.646	0.76 (0.47, 1.24) 0.270	0.232	
BMI						0.094
< 25	Reference (1.00)	0.68 (0.47, 0.97) < 0.05	0.39 (0.25, 0.60) < 0.001	0.48 (0.30, 0.76) < 0.01	< 0.001	
≥ 25	Reference (1.00)	0.87 (0.70, 1.07) 0.176	0.77 (0.61, 0.97) < 0.05	0.69 (0.52, 0.90) < 0.01	< 0.01	

**TABLE 7:** Association between plasma pyridoxal 5′‐phosphate level and depression in different subgroups.

	Plasma pyridoxal 5′‐phosphate (nmol/L)					
	**Q1 (2.00–25.80)**	**Q2 (25.90–42.80)**	**Q3 (42.90–73.90)**	**Q4 (74.00–324.00)**	*p* for trend	*p* for interaction
Age						0.899
< 55	Reference (1.00)	0.91 (0.73, 1.13) 0.381	0.75 (0.59, 0.96) < 0.05	0.73 (0.56, 0.95) < 0.05	< 0.01	
≥ 55	Reference (1.00)	0.88 (0.64, 1.20) 0.424	0.79 (0.56, 1.11) 0.171	0.82 (0.59, 1.15) 0.245	0.171	
Gender						< 0.01
Male	Reference (1.00)	1.40 (1.03, 1.92) < 0.05	0.98 (0.70, 1.38) 0.910	1.14 (0.81, 1.60) 0.459	0.977	
Female	Reference (1.00)	0.73 (0.59, 0.92) < 0.01	0.70 (0.55, 0.90) < 0.01	0.62 (0.47, 0.82) < 0.001	< 0.001	
Race						0.533
Non‐Hispanic White	Reference (1.00)	0.87 (0.67, 1.13) 0.298	0.67 (0.49, 0.91) < 0.05	0.74 (0.54, 1.00) < 0.05	< 0.05	
Others	Reference (1.00)	0.97 (0.76, 1.24) 0.807	0.88 (0.68, 1.14) 0.318	0.83 (0.62, 1.10) 0.192	0.143	
Educational level						0.214
Less than high school	Reference (1.00)	0.76 (0.48, 1.23) 0.265	0.90 (0.55, 1.47) 0.680	1.16 (0.70, 1.92) 0.571	0.590	
High school	Reference (1.00)	0.99 (0.77, 1.28) 0.967	0.74 (0.55, 0.99) < 0.05	0.74 (0.54, 1.01) 0.060	< 0.05	
College graduate or above	Reference (1.00)	0.83 (0.61, 1.12) 0.213	0.69 (0.50, 0.96) < 0.05	0.63 (0.45, 0.87) < 0.01	< 0.01	
Marital status						0.381
Married/living with partner	Reference (1.00)	0.98 (0.76, 1.25) 0.848	0.77 (0.59, 1.02) 0.070	0.72 (0.54, 0.97) < 0.05	< 0.05	
Widowed/divorced/separated	Reference (1.00)	0.72 (0.51, 1.00) 0.050	0.82 (0.57, 1.19) 0.303	0.87 (0.61, 1.25) 0.446	0.417	
Never married	Reference (1.00)	1.08 (0.71, 1.64) 0.722	0.69 (0.43, 1.11) 0.129	0.72 (0.43, 1.21) 0.219	0.085	
The ratio of family income to poverty						0.489
< 2.34	Reference (1.00)	0.91 (0.73, 1.13) 0.382	0.87 (0.68, 1.11) 0.271	0.83 (0.64, 1.08) 0.157	0.129	
≥ 2.34	Reference (1.00)	0.91 (0.64, 1.31) 0.619	0.63 (0.42, 0.93) < 0.05	0.61 (0.41, 0.90) < 0.05	< 0.01	
BMI						0.841
< 25	Reference (1.00)	1.01 (0.69, 1.46) 0.975	0.90 (0.60, 1.34) 0.592	0.76 (0.50, 1.14) 0.187	0.160	
≥ 25	Reference (1.00)	0.89 (0.72, 1.09) 0.246	0.73 (0.58, 0.92) < 0.01	0.77 (0.60, 0.98) < 0.05	< 0.01	

The results revealed that in almost all subgroups, dietary intake of vitamin B6 at the Q2–Q4 levels exhibited associations with a lower OR for depression when compared with that at the Q1 level. In some subgroups, the associations were discovered to be nonsignificant. We determined that the dietary intake of vitamin B6 did not exhibit significant interactions with the stratified variables (all *p* for interaction > 0.05).

In most subgroups, the PLP level in plasma at the Q2–Q4 levels exhibited associations with a lower OR for depression when compared with that at the Q1 level, although the associations were nonsignificant for some subgroups. We found no significant interactions between plasma 5′‐pyridoxine phosphate level and the hierarchical variables (all *p* for interaction > 0.05), except for gender‐based subgroup analysis (*p* for interaction < 0.05).

## 4 Discussion

This population‐based study on US adults was conducted to determine whether the PLP level in plasma and dietary intake of vitamin B6 are associated with depression risk. We discovered inverse associations of these variables with depression risk; we also noted the maintenance of these associations when considering subgroups defined by age, marital status, educational level, and ethnicity, BMI, as well as the ratio of family income to the poverty level. Our research results indicate that multiple factors may influence the associations of the PLP level in plasma and dietary intake of vitamin B6 with depression risk. Overall, the present study underscores the significance of ensuring that individuals with elevated depression risk are consuming sufficient vitamin B6 through their diet and maintaining their plasma PLP level at an appropriate magnitude. However, future randomized controlled trials (RCTs) are necessary to confirm the effects of vitamin B6 supplementation on depression risk. We should aim for a healthy and balanced diet in terms of nutritional supplementation.

Depression, as mentioned, is engendered by the intricate interaction of biological, social, and psychological factors, and biological and physical mechanisms have been suggested that plausibly link depression to the PLP level in plasma and dietary intake of vitamin B6. Vitamin B6, which comprises pyridoxal, pyridoxamine, and pyridoxine, is involved in the metabolism of depression‐onset‐associated neurotransmitters, thus rendering it a possible mitigating factor for hormone‐related depression (Bender 1999). Moreover, vitamin B6 levels were demonstrated by previous research to constitute a major factor for determining brain serotonin levels, and serotonin deficiency engenders panic attacks and sleep deprivation (Bell and Nutt 1998). Vitamin B6 activates decarboxylase, which is the main enzyme needed for synthesizing catecholamine and serotonin, and serotonin deficiency has been shown by previously executed studies to exhibit an association with depression and anxiety (Albert, Vahid‐Ansari, and Luckhart 2014; Sánchez‐Villegas et al. 2009). One study reported an increased p53‐overexpressing colon cancer risk in individuals with low dietary intake of vitamin B6 (Schernhammer, Ogino, and Fuchs 2008). Pyridoxine 5′‐phosphate constitutes the active form of vitamin B6 and has assumed a crucial role in plasma homocysteine regulation; homocysteine level elevation constitutes a vascular disease risk factor (Merete, Falcon, and Tucker 2008). The development of depression may be positively affected by the accumulation of homocysteine (Pan et al. 2012). An inverse association has been determined between inflammation and the plasma level of pyridoxine 5′‐phosphate. Proinflammatory cytokines mediate depression because it is an inflammatory disease (Lang and Borgwardt 2013), and this finding can explain the association between depression risk and the plasma level of pyridoxine 5′‐phosphate. The mechanism underlying the association of the plasma level of pyridoxine 5′‐phosphate with inflammatory markers remains to be determined. One possibility is that the plasma level of pyridoxine 5′‐phosphate being low reflects the entry of this coenzyme into inflammatory sites (Albert, Vahid‐Ansari, and Luckhart 2014; Sakakeeny et al. 2012). As revealed in the literature, the degree of stimulation of serum transaminase carrier enzymes significantly depends on the body's level of PLP (Westerhuis and Hafkenscheid 1983). A higher plasma level of PLP and more dietary intake of vitamin B6 are associated with reduced depression risk. However, caution should be exercised when recommending fortified foods or supplements, particularly those containing single B vitamins, as there is a potential risk of unbalanced nutrient intake. Nutritional scientists emphasize the importance of a healthy and balanced diet that cannot be replaced by supplements. Future research, including RCTs, is needed to explore the effects of vitamin B6 supplementation on depression and to provide clearer guidance on its clinical use.

Our study revealed that the PLP level in plasma and dietary intake of vitamin B6 in the higher quartiles were associated with reduced depression risk. Other studies have also reported this result for vitamin B6 intake (Kafeshani et al. 2020; Odai et al. 2020). Some research has indicated that elderly people's mental health is positively affected by appropriate B vitamin intake. One way of optimizing a person's B vitamin status and thereby helping to reduce their risk of depression is for them to regularly consume fortified foods (Moore et al. 2019). However, the results of one study did not indicate depression risk to be significantly associated with the dietary intake of vitamin B6 (Miyake et al. 2006). The reason for this discrepancy may be that this study's research sample was pregnant women, and during pregnancy, emotions are influenced by various factors, such as hormones, meaning that the effect of the dietary intake of vitamin B6 on depression is much weaker than the effects of other factors. Our results regarding dietary intake of this vitamin were found to be stable when we conducted subgroup analyses on the basis of age, gender, BMI, marital status, income, ethnicity, and educational level. However, this is inconsistent with the research findings of Kafeshani et al. (2020). Their results revealed no significant correlation of vitamin B6 consumption with depression or anxiety in men. This inconsistency may be due to the research population of their study being Iranian and, thus, due to the influence of ethnicity. When further exploring the relationship between plasma PLP and depression, the results showed that regardless of subgroup analysis for age, race, and income, the results were stable. However, in subgroup analysis based on gender, there was a statistically significant association between plasma PLP and depression in the female population, while not in males. The reason is likely that women are more susceptible to the influence of hormones compared to men. According to reports, women with hormone‐related depression lack vitamin B6 in their bodies and are associated with discomfort symptoms (Hasler et al. 2015).

Our research's strengths are multifold and are outlined as follows: First, we are, according to our literature review, the first to probe the associations of depression risk with the PLP level in plasma and dietary intake of vitamin B6 in the US population, in addition to being the first to probe the linear relationship of the PLP level in plasma with depression risk. Second, we applied our sampling design to obtain data from the NHANES datasets, which are well‐characterized and representative of the general US population, ensuring that the findings would be generalizable. Third, we used a restricted cubic spline to evaluate the linear relationships between the PLP level in plasma and dietary intake of vitamin B6 with depression. This spline is known to be suitable for determining nonlinear relationships; thus, we could obtain a favorable approximation of the true exposure–outcome relationships. Nevertheless, our study does have several limitations. First, we implemented a cross‐sectional design using health data from a single time point, and we, therefore, could not make causal inferences. In addition, given the significant differences in variables between individuals with high and low vitamin B6 intake, caution is warranted when interpreting the data. While we observed associations between higher vitamin B6 intake and reduced depression risk, correlation does not imply causation, and residual confounding factors may still be present.

## 5 Conclusions and Future Directions

The present results demonstrate associations between more dietary intake of vitamin B6 and higher plasma levels of PLP with lower depression risk. In addition, we obtained evidence of the aforementioned associations. The findings can offer insights into how vitamin B6 nutrition can possibly be leveraged to reduce a person's risk of depression and improve their health. Our findings must be validated through more multicenter, prospective, and large‐scale studies.

## Author Contributions

**Jinhong Lu**: conceptualization, data curation, formal analysis, funding acquisition, investigation, methodology, resources, software, supervision, validation, visualization, writing–original draft, writing–review and editing. **Guizhi Luo**: data curation, investigation, project administration, supervision. **Huina Mao**: data curation, resources, software. **Yulei Tan**: resources, software.

## Consent

Following the Declaration of Helsinki, all individuals participating in the NHANES gave their written informed consent.

## Conflicts of Interest

The authors declare no conflicts of interest.

### Peer Review

The peer review history for this article is available at https://publons.com/publon/10.1002/brb3.70128.

## Data Availability

The datasets analyzed during the current study are publicly available for download from the National Center for Health Statistics at the Centers for Disease Control https://www.cdc.gov/nchs/nhanes/index.htm.
